# Natural Preparations Based on Orange, Bergamot and Clove Essential Oils and Their Chemical Compounds as Antimicrobial Agents

**DOI:** 10.3390/molecules25235502

**Published:** 2020-11-24

**Authors:** Vlad Tiberiu Alexa, Camelia Szuhanek, Antoanela Cozma, Atena Galuscan, Florin Borcan, Diana Obistioiu, Cristina Adriana Dehelean, Daniela Jumanca

**Affiliations:** 1 Faculty of Dental Medicine, “Victor Babeş” University of Medicine and Pharmacy, Eftimie Murgu Sq. No. 2, 300041 Timişoara, Romania; vladalexa94@gmail.com (V.T.A.); szuhanek.camelia@umft.ro (C.S.); jumanca.daniela@umft.ro (D.J.); 2 Faculty of Agriculture, Banat’s University of Agricultural Sciences and Veterinary Medicine “King Michael I of Romania” from Timisoara, Calea Aradului No. 119, 300641 Timişoara, Romania; 3 Faculty of Pharmacy, “Victor Babeş” University of Medicine and Pharmacy, Eftimie Murgu Sq. No. 2, 300041 Timişoara, Romania; fborcan@umft.ro (F.B.); cadehelean@umft.ro (C.A.D.); 4 Faculty of Veterinary Medicine, Banat’s University of Agricultural Sciences and Veterinary Medicine “King Michael I of Romania” from Timisoara, Calea Aradului No. 119, 300641 Timişoara, Romania; dianaobistioiu@usab-tm.ro

**Keywords:** bergamot, orange, clove, eugenol, limonene

## Abstract

Since ancient times complementary therapies have been based on the use of medicinal plants, natural preparations and essential oils in the treatment of various diseases. Their use in medical practice is recommended in view of their low toxicity, pharmacological properties and economic impact. This paper aims to test the antimicrobial effect of natural preparation based on clove, orange and bergamot essential oils on a wide range of microorganisms that cause infections in humans including: *Streptococcus pyogenes*, *Staphylococcus aureus*, *Shigella flexneri*, *Candida parapsilosis*, *Candida albicans*, *Pseudomonas aeruginosa*, *Escherichia coli, Salmonella typhimurium and Haemophilus influenza*. Three natural preparations such as one-component emulsions: clove (ECEO), bergamote (EBEO), and orange (EOEO), three binary: E(BEO/CEO), E(BEO/OEO), E(CEO/OEO) and a tertiary emulsion E(OEO/BEO/CEO) were obtained, characterized and tested for antimicrobial effects. Also, the synergistic/antagonistic effects, generated by the presence of the main chemical compounds, were studied in order to recommend a preparation with optimal antimicrobial activity. The obtained results underline the fact that the monocomponent emulsion ECEO shows antimicrobial activity, while EOEO and EBEO do not inhibit the development of the analyzed strains. In binary or tertiary emulsions E(BEO/CEO), E(CEO/OEO) and E(OEO/ BEO/CEO) the antimicrobial effect of clove oil is potentiated due to the synergism exerted by the chemical compounds of essential oils.

## 1. Introduction

In medical practice, infectious diseases are a major health problem and the development of antibiotic-resistant bacteria is a challenge nowadays. In recent years there has been a growing interest in the use of natural products capable of replacing or being used to complement antibiotic treatment. Due to their availability, absence of side effects or toxicity, better biodegradability compared to available antibiotics and preservatives, essential oils (EOs) have aroused the interest of researchers [1,2]. EOs are complex natural mixtures of volatile secondary metabolites, isolated from plants by hydrodistillation. They have been shown to be biocids against a wide range of organisms, such as bacteria, fungi, viruses, protozoa, insects and plants [3,4,5,6].

EOs can be used in medicine and pharmacy as such or in the form of natural preparations with increased pharmacological activity. The formulation of natural preparations based on EOs influences their biological activity. In scientific research, different formulas of incorporation, encapsulation of EOs in natural preparations have been experimented. The most recently tested natural preparations based on EOs with antimicrobial activity refer to monodispersed essential oil-in-water (O/W) emulsions [7,8,9,10], emulsions with sodium starch octenylsuccinate [11], octenyl succinic anhydride (OSA)-modified gum Arabic (GA) emulsions [12], emulsions stabilized with zein colloid particles [13], nanoemulsions containing essential oils [14], emulsions stabilized by zein-pectin composite nanoparticles [15], micro and nano-encapsulation of EOs [16], gum-based coatings incorporated with EOs [17,18], cellulosic pads, amended with emulsions containing essential oils [19], electrospun nanofibers of poly(vinyl alcohol) and chitosan-based emulsions with EOs [20], chitosan-based materials [21], microencapsulation in sodium alginate and chitosan [22], EOs nano- and micro-sized formulations based on glycerol-plasticized cassava starch (TPS) [23].

Citrus oils have attracted the attention of researchers in recent years because they can be obtained as active ingredients from juice industry byproducts. Orange or bergamot oils are extracted using hydrodistilatilon from the peels after juice extraction, being a secondary raw material that can be used efficiently in the context of the circular economy. The antimicrobial activity of the orange essential oil (OEO) was evaluated and it exhibited significant inhibitory effects against many bacteria [24,25,26,27]. Obidi O. et al., highlighted that the Gram-positive bacteria were susceptible to the OEO with inhibitory concentration ranging from 0.05 mg/mL to 1.65 mg/mL [24]. Kirbaslar G. et al., tested the antimicrobial activity of lemon, grapefruit, bergamot, bitter orange, sweet orange and mandarin peel oils on different microorganism including: *Pseudomonas aeruginosa* ATCC 27853, *Staphylococcus aureus* ATCC 6538, *Escherichia coli* ATCC 11230, *Candida albicans* ATCC 10231 [25]. The Citrus peel oils showed strong antimicrobial activity against the Gram (+) and Gram (−) bacteria and the fungi cultures studied [26,27].

Other studies have focused on the antibacterial potential of bergamot essential oil (BEO). In a recent study, the group of Kokina et al. highlighted the effect of BEO against *Staphylococcus aureus* ATCC 25923 and *S. Typhimurium* ATCC 14028 [28], while Lazarotto et al. and Nashad et al. reported the inhibitory effect of BEO against *Pseudomonas aeruginosa, Escherichia coli, Staphylococcus epidermidis and Streptococcus pyogenes* [29,30]. Not least, the antifungal activity of BEO against clinically relevant Candida species was reported by Romano et al. [31].

Clove essential oil (CEO) represents one of the EOs with recognized antibacterial activity and wide applicability in medical practice for the complementary treatment of infections caused by gram positive or negative bacteria and fungi. Previous studies highlighted the multispectral antimicrobial activity of CEO and their main chemical compounds [13,17,32,33,34].

Our previous study highlighted the antibacterial role of essential oil-based emulsions on *Streptoccocus mutans*, as well as the synergistic effects generated by binary and tertiary mixtures [35].

This paper aims to test the antimicrobial effect of natural preparations based on clove, orange and bergamot essential oils on a wide range of microorganisms that cause infections in humans including: *Streptococcus pyogenes, Staphylococcus aureus, Shigella flexneri, Candida parapsilosis, Candida albicans, Pseudomonas aeruginosa, Escherichia coli, Salmonella typhimurium and Haemophilus influenza*. Also, the synergistic/antagonistic effects, generated by the presence of the main chemical compounds, were studied in order to recommend a preparation with optimal antimicrobial activity.

According to our knowledge, this is a first study that refers to the possibility of using natural preparations such as binary and tertiary emulsions containing essential oils of OEO, CEO and BEO as antimicrobial agents on a wide range of microorganisms, emphasizing at the same time the synergistic/antagonistic effects generated by the presence of the main chemical constituent compounds of EOs.

The choice of these EOs was made taking into account scientific, but also economic considerations. CEO is recognized and used for its antimicrobial properties. Our goal is to study the possibility of potentiating its effects by combining it with citrus oils. On the other hand, considering the huge quantities of citrus peels obtained from juice processing, the possibility of capitalizing them in the form of EOs with antimicrobial activity and applications in medicine and pharmacy was studied.

## 2. Results and Discussion

### 2.1. Characterization of Natural Preparations

The natural preparations based on EOs were obtained in the form of direct emulsions oil-in-water (O/W) in which the discontinuous phase is the oil globules dispersed in the aqueous phase. The composition of obtained emulsions is presented in Table 1.

The study of the CEO, OEO and BEO composition has been previously reported by our group [35]. CEO content eugenol as main component (80.11%) and eugenol acetate (13.54%). D-limonene represents 97.93% of OEO composition and 49.38% of BEO composition. Other minor components are: β-myrcene (1.52%, in OEO), α-pinene (32.63%, in BEO), *o*-cymene (3.82%, in BEO), 4-carene (6.35%, in BEO), β-linalool (3.02%, in BEO) [35]. Other studies have reported D-limonene as the main component in OEO and BEO [36,37,38,39] and eugenol in CEO [40].

The natural preparations obtained were analysed using Zetasizer parameters in order to detect the particle sizes and their electric charge at the surface. The analysed parameters: mean particle size (nm), polydispersity index (PDI), Zeta-potential (ζ-Potential) (mV) are presented in Table 2.

Mean particle size (nm) of natural preparations range between 180.6–624.3 nm falling into the category of emulsion and nanoemulsions. According to Gupta et al., nanoemulsions are characterized by particle sizes between 20–500 nm, thermodynamically instability and kinetic stability and polydispersity (<10–20%) [41]. The physical stability of emulsion systems is characterized by the mean particle size that describes the average size of the dispersed oil droplets and PDI that measures the size distribution and stability of droplet sizes in the emulsion [42]. Nanoemulsions are colloidal dispersions consisting of two immiscible solvents—oil (globules) and water (liquid)—in which one is dispersed in the other with the help of a surfactant that stabilizes the emulsion. Nanoemulsions are promising nanocarriers widely used in drug delivery, which help in improving the biodistribution of drugs and minimizing toxicity [3]. Uni-populational emulsions were obtained in the case of the samples EBEO (277.3 nm), EOEO (320.2 nm) and E(BEO/OEO) (315.5 nm), while the other samples present two particle populations with different ratios. According to Borcan et al., a polydisperse system based on polyurethane structures displays improved release kinetic due to the heterogeneity of that sample and it can assure an almost constant concentration of the active agent in the case of a prolonged release [43]. On the other hand, Danaei et al. consider that a safe formulation based on stable and efficient nanostructures requires the preparation of a monodisperse (uni-population) delivery system [44].

PDI values are between 0 and 1, where 0 is specific to homogeneous systems, and 1 for highly heterogeneous ones. Zeta-potential (ζ-Potential) is an indicator of emulsion stability used to measure the net surface electrical charge on the emulsion droplets [42]. The Zeta potential values are between −24.31:−19.72 mV, natural preparations obtained falling into the category of delicate dispersions. In general, colloidal solutions have a high tendency to form clusters/clusters–agglomerations. This agglomeration trend can be estimated using Zeta potential values: stable systems have Zeta potential values between −30 and −100 mV, where 0 mV represents the maximum agglomeration and precipitation. The decrease in the ζ-potential values would assist for the long-term stability of the emulsions [10].

### 2.2. Antimicrobial Activity of Natural Preparations

**Figure 1:**
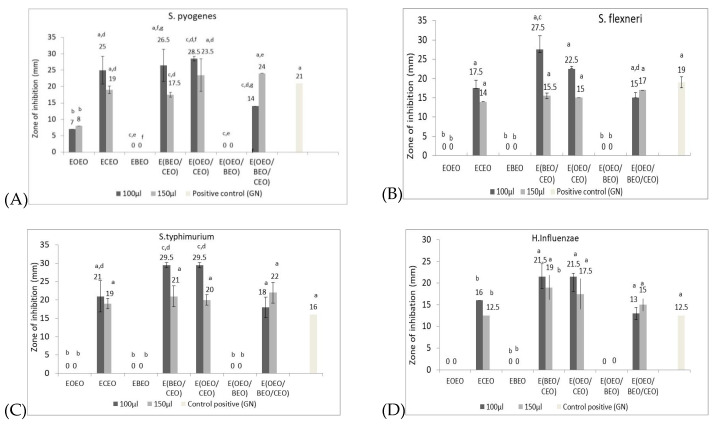

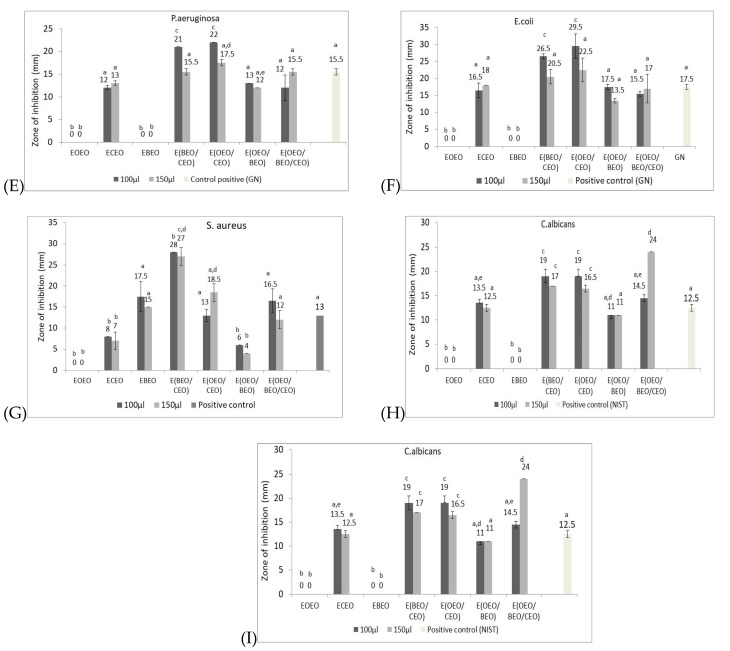
Antimicrobial activity of natural preparations expressed as Zone of inhibition (mm). (**A**) *S. pyogenes*, (**B**) *S. flexneri,* (**C**) *S. typhimurium;* (**D**) *H.influenzae;*; (**E**) *P.aeruginosa*; (**F**) *E.coli*; (**G**) *S.aureus;* (**H**) *C.albicans;* (**I**) *C.parapsilopsis* (different letters in columns indicate significant differences between values according to the T test, *p* < 0.05).

Figure 1 shows the values of the inhibition zones of analysed strains in the presence of the natural preparations, presented as an average of three determinations, as well as the standard deviation for emulsions and positive control.

*Streptococcus pyogenes* is a species of Gram-positive, aerotolerant bacterium from the genus Streptococcus [45]. It can cause a variety of diseases such as streptococcal pharyngitis, rheumatic fever, rheumatic heart disease, and scarlet fever [46]. *Shigella flexneri* is a species of Gram-negative bacteria in the genus *Shigella* that can cause diarrhea in humans [47]. *Shigella* are closely related to *Escherichia coli*, but can be differentiated from *E. coli* based on pathogenicity, physiology and serology [48].

From the data shown in Figure 1A it is observed that the ECEO exerts a greater inhibitory effect on *S. pyogenes* (25 mm inhibition area, for a concentration of 100 µL) higher compared to the positive control gentamicin (21 mm diameter inhibition). The diameter of the inhibition zone for EOEO is 7–8 mm depending on the applied concentration, and for EBEO the effect of inhibiting the micellar development of *S pyogenes* is zero, regardless of the applied concentration. The application of binary solutions E(OEO/CEO) leads to the potentiation of the capacity to inhibit micellar development (28.5 mm, respectively 23.5 mm), synergistic activity developed by associating the active principles from the two EOs, eugenol from CEO and D-limonene from OEO, respectively.

The association of E(BEO/OEO) does not show antibacterial effect against *S. pyogenes*, regardless of the component concentration. This behavior can be generated by the antagonistic effects of the OEO and BEO active principles. BEO contains in addition to D-limonene, in significant concentration, α-pinene (32.63%), but also other minority compounds such as: *o*-cymene (3.82%), 4-carene (6.35%) and β-linalool (3.02%), probably responsible for the antagonistic action on *S. pyogenes*. Also, the E(BEO/CEO) association decreases the antibacterial effect of *S. pyogenes* compared to the ECEO. The major compound in CEO is eugenol, responsible for the effects of inhibiting micellar growth, effect diminished by the addition of BEO, especially when increasing its concentration (17.5 mm diameter of the inhibition zone for a concentration of 150 µL emulsion).

The tertiary emulsion, obtained by combining BEO, without effect on *S. pyogenes*, with E(OEO/CEO) conducted to an increase in the diameter of the inhibition zone when 150 µL was applied (24 mm), and higher than the positive control (21 mm).

Statistically significant differences, compared to the positive control, were registered for the monocomponent variants of EOEO and EBEO type, regardless of concentration, respectively for the binary mixtures of E(OEO/BEO) type. For CEOs in the one-component or two-component version, E(BEO/CEO) 150 µL, E(OEO/CEO) 100 µL, respectively three-component E(BEO/CEO/OEO), 100 µL, there were no statistically significant differences compared to control consisting of gentamicin.

The experimental results highlight that the optimal formula, which generates maximum inhibition effect against *S. pyogenes*, is E(OEO/CEO). Previous studies highlighted the antimicrobial effect of analysed EOs against *S. pyogenes*. Wijesundara and Rupasinghe tested in vitro the antibacterial effect of clove essential oil on two *S. pyogenes* strains (ATCC 19615 and ATCC 49399) [49]. The results highlighted that CO exhibited an important antibacterial activity, the minimum inhibitory concentration (MIC) concentrations ranged between 0.25–1.0 mg/mL and the minimum bactericidal concentration (MBC) were 0.5–2 mg/mL.

The application of one-component emulsions on *S. flexneri*, shows a response similar with the one obtained against *S. pyogenes* (Figure 1B). The ECEO is found to have an inhibitory effect (17.5 mm for 100 µL applied emulsion), but lower compared to the positive control gentamicin 10 mg (19 mm), while EOEO and EBEO have no effect on *S. flexneri*, regardless of the concentration applied.

It should be noted that even if in the one-component variant EOEO and EBEO do not register any inhibitory effect, by associating with the ECEO, the chemical components, especially D-limonene present in both OEO and BEO and eugenol in CEO, exert synergistic effects and increase the inhibition effect of ECEO. The E(BEO/CEO) emulsion (100 µL) has a diameter of the inhibition zone of 27.5 mm, respectively 22.5 mm in the E(OEO/CEO), over the diameter of the positive control, gentamicin (19 mm) and ECEO (17.5 mm). The tertiary emulsion E(OEO/BEO/CEO), has antibacterial activity comparable to that exerted by the one-component ECEO emulsion (17.5 mm, respectively 14 mm for doses of 100 µL and 150 µL emulsion). The obtained results confirms previous data regarding the strong inhibitory effect against S. flexneri of ECEO (about 23 mm for diameter of inhibition zone) [50].

From a statistical point of view, it can be seen that ECEO-based emulsions do not show significant differences compared to the positive control (gentamicin), while the results obtained for EOEO and EBEO, alone or in a mixture, are significantly different from those of positive control.

The optimal emulsion recommended for a maximum degree of inhibition of micellar development of *S. flexneri* are the E(OEO/CEO) binary mixtures and E(BEO/CEO) at a level of 100 µL.

*Salmonella typhimurium* is a pathogenic Gram-negative bacteria predominately found in the intestinal lumen and causes gastroenteritis in humans and other mammals [51,52].

The profile of antibacterial activity on *S. typhimurium* when one-component, binary or tertiary emulsions based on EOEO, EBEO and ECEO were applied is similar to the effect exerted by the mentioned emulsions on *S. flexneri* (Figure 1C). An important inhibition rate is observed when the one-component emulsion based on ECEO (21, respectively 19 mm inhibition area compared to 16 mm for the positive control) was used, while EOEO and EBEO have no effect regardless of the applied concentration. The use of E(BEO/CEO) binary mixtures respectively E(OEO/CEO) potentiates the antibacterial effects due the synergism created by the main chemical components (eugenol in CEO and D-limonene in OEO and BEO). Binary mixtures exert an antibacterial effect on *S. typhimurium* higher than ECEO monocomponent emulsion and over the gentamicin (inhibition zone 21–29.5 mm for E(BEO/CEO), respectively 29.5–20 mm for E(OEO/CEO), depending on the amount of emulsion applied. The tertiary mixture E(OEO/CEO/BEO) exerts an important inhibitory effect of the *S. typhimurium* mycelium (22 mm and 18 mm, respectively), higher than positive control (16 mm), but smaller than binary mixtures. Values comparable to the positive control (gentamicin), from a statistical point of view, are recorded for ECEO, E(BEO/CEO) and E(OEO/CEO) (150 µL), respectively for the tertiary mixture E(OEO/BEO/CEO), regardless of concentration.

*Haemophilus influenzae* is a Gram-negative, coccobacillary, facultatively anaerobic capnophilic pathogenic bacterium of the family Pasteurellaceae [53]. In infants and young children, *H. influenzae* type b causes bacteremia, pneumonia, epiglottitis and acute bacterial meningitis. On occasion, it causes cellulitis, osteomyelitis, and infectious arthritis. It is one cause of neonatal infection [54].

The antimicrobial profile of the emulsions against *H. influenzae* has the same characteristics as the one obtained for *Salmonella typhimurium*. EOEO and EBEO monocomponent emulsions have no activity, only the ECEO has an antibacterial effect. In bicomponent emulsions, the addition of OEO and CEO leads to increased antimicrobial activity, the same effect being observed in the case of tertiary emulsion E(OEO/CEO/BEO) (Figure 1D).

Figure 1E,F show the antibacterial effect of the analyzed emulsions on *P. aeruginosa and E. coli. Escherichia coli* is a Gram-negative, facultative anaerobic, rod-shaped, coliform bacterium of the genus *Escherichia* that is commonly found in the lower intestine of warm-blooded organisms [55,56]. Most *E. coli* strains are harmless, but some serotypes can cause serious food poisoning in their hosts, and are occasionally responsible for food contamination incidents [57,58].

*Pseudomonas aeruginosa* is a common encapsulated, Gram-negative, rod-shaped bacterium. P. aeruginosa is a multidrug resistant pathogen recognized for its ubiquity, its intrinsically advanced antibiotic resistance mechanisms, and its association with serious illnesses—hospital-acquired infections such as ventilator-associated pneumonia and various sepsis syndromes [59].

It is observed that ECEO is responsible for the antibacterial activity, but the inhibitory capacity of the one-component emulsion is lower in this case compared to the effect exerted on the other analyzed gram positive or negative bacteria. The diameter of the inhibition zone is smaller than the positive control in the case of *P. aeruginosa* (12 mm for the application of 100 µL emulsion, respectively 13 mm for 150 µL, compared to gentamicin 15.5 mm). The diameter of the inhibition zone does not differ statistically significantly from the positive control (16.5/18 mm compared to 17.5 mm in the case of gentamicin). Statistically significant differences compared with the positive control (gentamicin) are noted for one-component emulsions EOEO and EBEO (without effect), respectively binary emulsions E(BEO/CEO) (100 µL) and E(OEO/CEO) (100 µL) (a potentiated effect compared to the control), both in the case of *P. aeruginosa* and *E. coli.*

The association of binary components E(OEO/CEO), E(BEO/CEO) leads to the potentiation of the inhibition effect, which increases significantly compared to the positive control, especially when applying 100 µL emulsion (21/22 mm for *P. aeruginosa* and 26, 5/29 mm for *E. coli*). This confirms the synergistic effect exerted by associating the active principles of CEO (eugenol) with the main terpene components of OEO and BEO (D-limonene, α-pinene). The same effect was reported by Cho et al., where an antibacterial effect of clove oil against *E. coli* was observed for 600 µg/mL. Other studies reported the inhibitory effect of eugenol, the main chemical compound of clove oil [3,4,5].

The potentiation of the antibacterial effect by combining EOs in binary preparations is also reflected in the antibacterial effect exerted in the case of E(OEO/BEO) emulsions. If in the monocomponent variant EOEO and EBEO did not inhibit the micellar development of *P. aeruginosa* and *E. coli*, applied in binary formula proved to be effective, but the effect is lower compared to the positive control.

The antibacterial efficiency of plant compounds depends on several factors such as characteristics of microorganism analyzed, characteristics of the plant taken into research and the chemical properties of the solution obtained and tested. Plant-derived compounds (biologically active substances, generally secondary metabolites, given the fact that they occur as an intermediate or end products of secondary plant metabolism) could exhibit a direct antibacterial activity and/or an indirect activity. Because essential oils represent complex mixtures of compounds, belonging to different chemical classes that share some general mutual characteristics, such as polarity and/or volatility, synergistic or antagonistic interactions between two or more main components, in which one enhances the effect of the other and together they act more efficiently than as individual agents, are more than frequent, becoming the main motivation behind many researchers. [60,61].

As our results in Figure 1E,F present, the Gram-negative strains (*P. aeroginosa* and *E. coli*) were more sensitive to the synergistic effect of the tested emulsions than Gram-positive strains. There are several possible causes, the most often cited one being the particularities of the cell wall and cell membrane [62]. Hemaiswarya et al., noticed synergistic interactions of eugenol from *Eugenia* plant aromatics with 10 different hydrophobic and hydrophilic antibiotics in case of five Gram-negative bacteria. Synergism occurred due to ability of eugenol to increase the permeability of cell membrane, by reducing the density of the lipids and resulting in a permeable membrane [62]. We considers that most likely the same think occurred with our emulsions, one component acting perhaps as an agent that modified the permeability of the cell membrane, while another one acted as an antimicrobial agent, once it got inside the pathogenic cell.

*Staphylococcus aureus* is a Gram-positive bacterium frequently found in the upper respiratory tract and on the skin [63]. *S. aureus* can also become an opportunistic pathogen, being a common cause of skin infections including respiratory infections such as sinusitis, abscesses, and food poisoning [64].

In the case of *S. aureus* (Figure 1G), when applying the one-component emulsions, the maximum inhibition effect is noticed for EBEO (diameter of the inhibition zone between 15–17.5 mm, depending on the amount applied), this being higher than the effect exerted in the case of gentamicin treatment. Inhibition of *S. aureus* development, although smaller compared to the positive control, is observed for ECEO emulsion (diameter of the inhibition zone between 7–8 mm, depending on the amount applied), the value differences being statistically significant. EOEO does not exert antibacterial effect against *S. aureus*, regardless of the amount applied. The use of binary emulsions of E(BEO/CEO) leads to the increase of inhibition potential against *S. aureus*, the registered value being statistically significant both compared to the positive control and to the one-component emulsions. The inhibition zone of E(OEO/CEO) mixture, was not statistically significantly different from those recorded for gentamicin, while the E(OEO/BEO) emulsion leads to a lower effect than the control (statistically significant differences) and comparable to the effect produced by the one-component ECEO.

*Candida albicans* is an opportunistic pathogenic yeast that is a common member of the human gut flora, found in 40 to 80% of normal human beings as commensal in gastrointestinal tract [65]. It is usually a commensal organism, but it can become pathogenic under a variety of conditions. It is a commonest cause of candidiasis (moniliasis). Other most common isolate of Candida species are *Candida tropicalis*, *Candida parapsilosis*, and *Candida glabrata* [66,67].

*Candida parapsilosis* is a fungal species of yeast that has become a significant cause of sepsis and of wound and tissue infections in immunocompromised people. Unlike *Candida albicans* and *Candida tropicalis, C. parapsilosis* is not just a human pathogen, having also been isolated from non-human sources such as domestic animals, insects and soil [68]. *C. parapsilosis* was the second most common Candida species isolated from normally sterile body sites of hospitalized patients. It accounted for 15.5% of *Candida* isolates in North America, 16.3% in Europe, and 23.4% in Latin America, outranked only by *C. albicans* (51.5%, 47.8%, and 36.5%, respectively) [68].

The antifungal activity of the analyzed emulsions against *Candida albicans* and *Candida parapsilopsis* is shown in Figure 1H,I. It is observed that the profile of the micellar growth in the case of the two analyzed fungi is similar, higher antifungal effect of the one-component emulsion ECEO being observed regardless of the concentration tested. EOEO and EBEO do not exert antifungal effect on the investigated strains. The association of E(OEO/CEO) and E(BEO/CEO) leads to an increase of the antifungal activity against *C. albicans* (the diameter of the inhibition zone for E(BEO/CEO) depending on the concentration, 19/15 compared to 13.5/12.5 in the case of ECEO, respectively 19/16.5 for E(OEO/CEO), which recommends the use of these binary solutions in the control of *C. albicans*.

Binary solutions applied on *C. parapsilopsis* compared to the ECEO monocomponent emulsion, provided a similar antifungal effect, there are no statistically significant differences, a slight increase of the inhibition zone being registered in the case of E(OEO/CEO) 100 µL (27 mm compared to 20 mm in CEO).

The findings of the study conducted by Pinto et al., indicated that CEO has potential as a therapeutic option against fungi that are pathogenic to humans including *C. albicans, C. tropicalis* and *C. parapsilosis* [32]. In the presence of binary emulsion E(BEO/OEO), the different behaviour of *C.albicans* and *C. parapsilopsis* is observed. If in the case of the first fungal strain an antifungal effect is observed as a result of the synergism created by the constituent compounds, in the case of *C. parapsilopsis* this is not highlighted (Figure 1H,I). The different mechanism of action can be explained by: (i) different modification of active sites on fungal cell, (ii) different capacity to inhibit the enzymes, (iii) increase/decrease of membrane permeability [60]. In the case of the two fungi studied, we notice the maximum inhibition potential exhibited by the tertiary emulsion E(CEO/BEO/OEO), when applying 100 µL.

From a statistical point of view, significant differences compared to the positive control, are registered for EOEO, EBEO, E(OEO/CEO), E(BEO/CEO) and E(OEO/BEO/CEO) 150 µL emulsions. In the treatment applied to *C. parapsilopsis*, statistically undifferentiated results compared to the control were recorded for E(OEO/BEO), ECEO (150 µL),E(OEO/CEO) (150 µL) and E(BEO/CEO) (150 µL).

**Figure 2:**
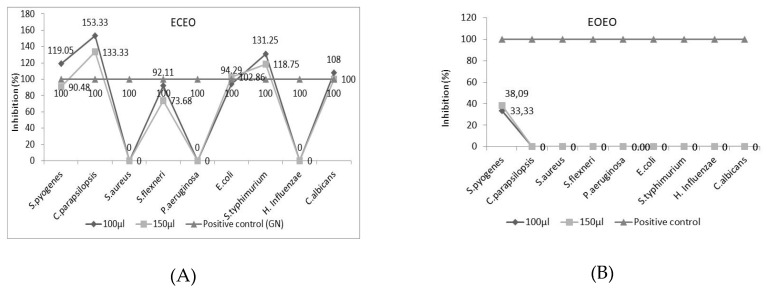

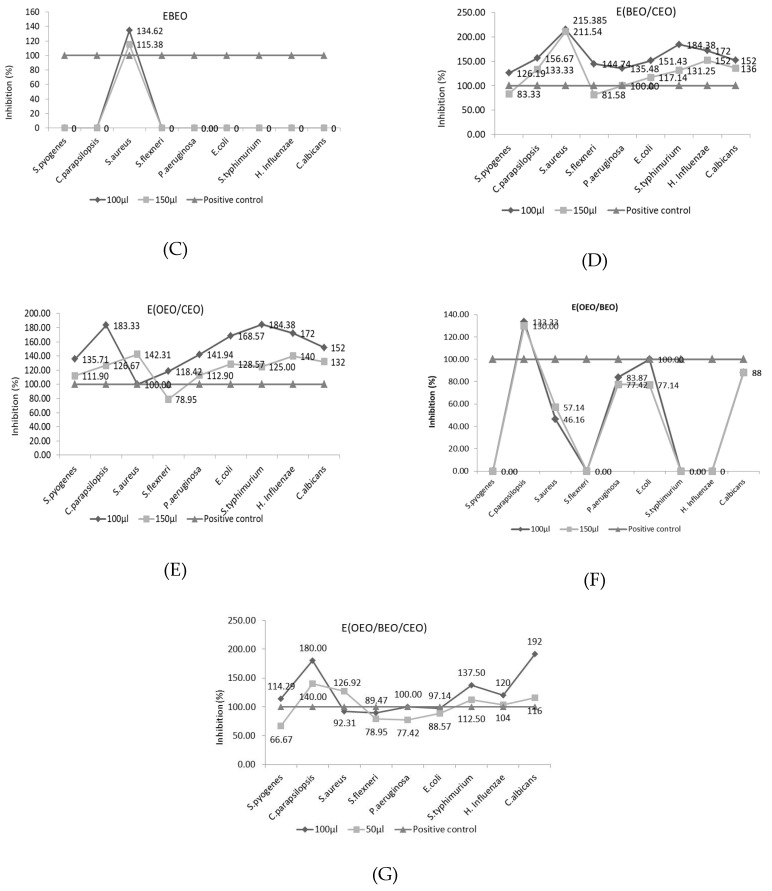
Inhibition rate (%) of natural preparations (**A**) ECEO, (**B**) EOEO, (**C**) EBEO, (**D**) E(BEO/CEO), (**E**) E(OEO/CEO), (**F**) E(OEO/BEO), (**G**) E(OEO/BEO/CEO).

Figure 2 shows the Inhibition rate (%) calculated according to Equation (1) in Section 3.3 It is observed that ECEO shows an inhibitory effect over the positive control in the case of *C. parapsilopsis* (133.33% and 153.33% depending on the applied concentration), as well as in the case of *S. typhimurium* (118.75% and 131.25 %). In both cases higher values of inhibition being registered when 100 µL emulsion was applied (Figure 2A). Natural preparations based on EOEO or EBEO in the one-component variant have a reduced antimicrobial spectrum. EOEO was found to be active only for *S. pyogenes*, with a lower degree of inhibition than gentamicin (33.33%, respectively 38.09% depending on the amount applied), while EBEO is effective on *S. aureus* with an inhibition rate of 115.38% and 134.62%, respectively, depending on the amount applied, compared to the positive control (Figure 2B,C). The association of EOs in binary mixtures of type E(BEO/CEO), E(OEO/CEO) and E(OEO/BEO) leads to synergistic effects of the active principles from the monocomponent variants and the potentiation of the microbial inhibition by the natural preparations.

Applying the E(BEO/CEO) emulsion, an inhibition rate of over 100% is registered for most of the analyzed microbial species (Figure 2D). The synergistic effects exerted in the binary variants determine the activation of the antibacterial properties of the active principles existing in latent state in the monocomponent variants. Thus, if for the monocomponent variants ECEO and EBEO no inhibitory effect was registered, in the case of *P. aeruginosa and H. influenzae*, it is observed that applying the binary mixture E(BEO/CEO), the inhibition rate is 100% and 135.48%, depending on the amount applied, compared to gentamicin (100% inhibition rate) for *P. aeruginosa*, respectively 152% and 172% for *H. influenzae*.

The same effect is observed for E(OEO/CEO) emulsion which is noted to have maximum efficiency, with an inhibition rate over 100%, against all microbial species analyzed (Figure 2E). The inhibitory effect varies (in case of application of 100 µL emulsion): *S. aureus < S. flexneri < S. pyogenes < P. aeruginosa < C. albicans < E. coli < H. influenzae < C. parapsilosis < S. typhimurium.* When 150 µL emulsions were used, the antimicrobial effect varies: *S. flexneri < S. pyogenes < P. aeruginosa < S. typhimurium < C. parapsilosis < C. albicans < H. influenzae < E. coli < S. aureus.*

The ternary emulsion E(OEO/CEO/BEO) shows antimicrobial effects on all studied species, values of inhibition rate higher than 100% being registered against *S. pyogenes, C. parapsilosis, S. typhimurium, H. influenza, C. albicans*, regardless of the amount applied (Figure 2G).

### 2.3. The Minimum Inhibitory Concentration (MIC) of Chemical Compounds, EOs and Natural Preparation

Considering the different potential of the emulsions depending on their chemical composition, the determination of MIC, both for emulsions, EOs and and of the main chemical compounds was performed. The MIC is defined as the lowest concentration of the compounds to inhibit the growth of microorganisms. The MICs (µL/100 mL) values of main chemical compounds (eugenol, limonene and alfa-pinene), EOs: clove (CEO), orange (OEO), bergamot (BEO) and natural preparation are presented in the Table 3.

The MICs of eugenol, the main compound of CEO, was found to be 2 µL/100 mL, but the inhibition effect decreased with concentration for all tested strains. Given the composition of CEO (80.11% eugenol), it was expected, as proved by experimental values, that the MIC for CEO is close to the eugenol value (2 µL/100 mL), except for the values obtained for *Shigella flexneri* and *E. coli*, where the MIC is greater than 7 µL/100 mL. The results concerning the MIC for each of the standards tested correlate with the literature data. Pinto et al., 2009 reported an average of 6.6 µg/100 mL for volatile terpene compounds (thymol, *p*-cymene, γ-terpinene), and MIC = 120 µL/mL for eugenol, respectively, against *Escherichia coli* and 0.5 µL/mL against *Salmonella Typhimurium* [32]. Results regarding the antimicrobial activity of terpene compounds from essential oils have been previously reported [3]. Eugenol has proven antimicrobial activity against *Candida* spp. (*C. albicans*) and *S. aureus* and *Haemophilus* strains [3,69], while linalool is active against *E. faecalis, E. coli*, *K. pneumoniae, S. aureus, S. epidermidis, P. aeruginosa, C. albicans, B. subtilis, S. typhimurium* [3].

Kozics et al. reported a MIC of 0.5% (*w*/*v*) for CO against *P. aeruginosa, Candida albicans* and *C. parapsilopsis* [4]. Another study highlighted the inhibition potential of CO against *S. aureus and P. aeruginosa* with MIC’s that ranged from 2.5 to 5.0 mg/mL [5].

In the one-component eugenol-based emulsion ECEO the antimicrobial effect at a similar oil concentration is diminished (MIC > 40 µL/100 mL, excepting *Haemophillus influenzae* (MIC = 40 µL/100 mL) and can be explained by possible inhibitory effects generated by the excipients used in the preparation of the emulsions. 

d-Limonene represents 97.93% of the OEO composition and 49.38% of BEO. The MICs for limonene were found to be 2 µL/100 mL, excepting *Streptococcus pyogenes* for which the MIC value was 7 µL/100 mL. The development of the fungus is different depending on the concentration and tested strains. Thus, for *S. aureus, Haemofilus influenzae* and *Candida albicans*, the inhibitory effect decreases with increasing tested concentration above the MIC, the value of 2 µL/100 mL being optimal, while for the other strains the antimicrobial effect increases with increasing tested concentration over the MIC. A similar profile to that of limonene is registered when OEO and BEO were tested, with some modifications caused by the presence of other minor components that act differently depending on the strain studied. Thus, if limonene has antimicrobial effect on *Pseudomonas aeruginosa* (MIC 2 µL/100 mL), OEO does not inhibit the micellium growth, even at 7 µL/100 mL. The same trend was recorded for limonene and BEO against *Streptococcus pyogenes.*

One-component emulsions have been shown to be less effective in terms of antimicrobial effect compared to standards and constituent oils, having MIC > 140 µL/100 mL. The same behavior is registered in the case of binary emulsions E(OEO/BEO), while the association of E(OEO/CEO) and E(BEO/CEO) (MIC = 40 µL/100 mL, excepting *Escherichia coli* MIC > 140 µL/100 mL) leads to synergistic effects that enchaced the antimicrobial activity against tested strains. The tertiary emulsion E(BEO/CEO/OEO) has a MIC of 40 µL/100 mL for majority strains, excepting *Streptococcus pyogenes*, *Shigella flexneri* and *E. coli* (MIC > 140 µL/100 mL).

The data obtained regarding MIC are correlated with those obtained by the diffusion method and presented in the Section 2.2, confirming the antimicrobial effects of the binary E(OEO/CEO) and E(BEO/CEO) emulsions, as well as of the tertiary one, on most of the studied strains.

## 3. Materials and Methods

### 3.1. Obtaining and Characterization of Natural Preparations

The natural emulsions were obtained based on EOs from CEO, BEO and OEO (SC SOLARIS PLANT SRL, Bucharest, Romania) using the method described in our previous paper [35]. The emulsifier (lecithin) was dissolved in water and the EO was mixed using a VCX130 PB Ultrasonic Processor (130 Watt, Frequency 20 kHz, Sonics & Materials Inc., Newtown, CT, USA), for 10 min at an amplitude of 98%. The monocomponent emulsions (EOEO, EBEO, ECEO) were obtained by adding 1 mL of EOs in 19 mL water and 6 mg of lecithin (WalMart, Rogers, AR, USA) as emulsifier. Binary emulsions E(OEO/BEO), E(OEO/CEO), E(BEO/CEO) were obtained by adding one mL of each EOs, 6 mg lecithin and 18 mL water. The tertiary emulsion E(OEO/BEO/CEO) was prepared by adding 1 mL of each EOs, 6 mg lecithin and 17 mL water.

The particle sizes and their electric charge at the surface were determined using a Zetasizer instrument (Cordouan Technology, Cité de la Photonique, France). Mean particle size (nm), polydispersity index (PDI), and ζpotential (mV) were determined. The instrument consists of a size analyzer, known as the Vasco Particle Size Analyzer, and a Zeta potential analyzer, called the Wallis. For these determinations, the following measurement parameters were used: vat type (plastic, 380 to 780 nm long, visible spectrum), working temperature (23 °C), laser power (80 ± 5%), position DTC: UP, channel number (~450) and time range (15 ± 5 μs), data acquisition mode-continuous, with at least five measurements/sequence (statistical distribution) at medium resolution with Pade-Laplace analysis mode and Henry-Smoluchowski functions for the analysis of experimental data.

### 3.2. Microbial Strains

The used microbial strains were obtained from the culture collection of the Laboratory of Microbiology, Interdisciplinary Research Platform within Banat’s “King Michael I of Romania” University of Agricultural Science and Veterinary Medicine Timisoara. The analysed strains were: *Staphylococcus aureus* (ATCC 25923), *Streptococcus pyogenes* (ATCC 19615), *Escherichia coli* (ATCC 25922), *Pseudomonas aeruginosa* (ATCC 27853), *Shigella flexneri* (ATCC 12022), *Salmonella typhimurium* (ATCC 14028) and *Haemophillus influenzae* type B ATCC 10211 and the fungal strains were *Candida albicans* (ATCC 10231) and *Candida parapsilopsis* (ATCC 22019). The strains are maintained at −50 °C until the experiment assays were performed.

### 3.3. Disk Diffusion Method 

Emulsions were tested using the disk diffusion method for susceptibility testing, according to the Standard Rules for Antimicrobial Susceptibility Testing using Impregnated Disks. In vitro testing was performed in plates, containing microcomprimates with nystatin for the antifungal activity and gentamicin for the antibacterial activity as positive controls, commercial gentamicin discs (10 mg—Ref. E110712, BioMaxima, Lublin, Poland) and Nystatin (100 mg—Re. SD 025, HiMedia, Mumbai, India), alongside blank filter papers not impregnated as negative controls and filter papers impregnated with a known quantity of the tested samples. Blank filter paper had a diameter of 5 mm.

A 10^−2^ dilution of the fresh *C. albicans* and *C. parapsilopsis* culture and a 10^−3^ fresh gram positive and gram negative strains culture were used to perform the assay, an inoculum equivalent to a 0.5 McFarland standard. The Petri plates so seeded in which the positive, negative and sample emulsions were added, were incubated at 30 °C for *C. albicans* and *C. parapsilopsis* and 37 °C in case of the antibacterial testing, for 24–48 h. Tests were performed in triplicate.

The antimycotic and antibacterial effect of the natural preparations was achieved by measuring the diameter of the analysed culture inhibition zones (including the diameter of the disc—5 mm) in millimeters. Also, the inhibition ratio reported to the positive control (gentamicin/nystatin) and negative control (papers not impregnated) was calculated according the formula [36]:

% inhibition = 100 × [1 − (D_sample_ − D_positive_)/(D_negative_ − D_positive_)] (1)

where D negative—diameter of paper not impregnated (mm); D positive—diameter of entamicin/nystatin inhibition zone (mm) and D sample—diameter of inhibition zone of sample with inhibitor agent (mm).

### 3.4. Minimum Inhibitory Concentration (MIC) of Standards, EOs and Emulsions

The method of MIC determination based on the microbial mass loss by measurement of the optical density (OD) by spectrophotometry according ISO 20776-1:2019 and was described in our previous research [35].

The ATCC strains used were the same ATCC strains (Section 3.2.), revived by overnight growth in Brain Heart Infusion (BHI) broth (CM1135, Oxoid, Hampshire, U.K.) at 37 °C. It was tested the main chemical compounds of essential oils (eugenol, limonene and α-pinene), the clove essential oil (CO), orange oil (OO), bergamot oil (BO) and the 7 obtained emulsions presented in Table 1.

A fresh culture dilution at 10^−3^ was used to perform the analysis, with an inoculum equivalent to 1.5 × 108 CFU/mL (0.5 McFarland standard). The resulting dillutions were tested using a 96 SPL Cell Culture Plate (Cat.no. 31096, SPL Life Sciences Co., St. Petersburg, FL, USA) with a working volume of 200 μL. Using a Socorex Calibra digital 852 multichannel pipette (No. SOCO_13018, Socorex Isba SA, Ecublens, Switzerland), 100 μL of strain diluted suspension was placed in each well. The analyzed sample was applied directly inside each well, introducing 2, 5 and 7 µL/100 mL in case of EOs and standards and 40, 100 and 140 µL/100 mL, respectively, in case of the emulsions. 

In order to define the MIC the composition of the EOs and emulsions was considered. The quantities for each emulsion were determined after preliminary tests that indicated the MIC for each of the standards involved (limonene, eugenol and alfa-pinene), the values being in the rage of 2–7 µL/100 mL. For this motive the concentrations tested were 2, 5 and 7 µL/100 L. Subsequantly, these values obtained in the preliminary tests were used to calculate the necesary quantity for each essential oils, respectively emulsions, so as to contain aproximatly that specific standard concentration.

After the introduction of the samples, the plates were covered and incubated for 24 h at 37 °C. The growth of bacteria and fungi was determined by OD values at 540 nm using an ELISA reader (PR 1100 BioRad, Tallin, Estonia). The tests were performed in triplicate for all samples and the mean values were calculated and used subsequently. Simple strains suspensions in BHI were used as control.

### 3.5. Statistical Analysis

The mean values and standard deviations of all replicates were calculated using the Excel software. Differences between means were analyzed with a one-way ANOVA, followed by multiple comparison analysis using the T test. Differences were considered significant when *p*-values < 0.05.

## 4. Conclusions

The use of natural preparations such as emulsions and nanoemulsions represents a viable alternative to synthetic compounds currently used in antimicrobial control. The obtained results highlight the possibility to use some EOs obtained as by-products from the capitalization of citrus fruits (orange and bergamot) as antimicrobial agent against some pathogenic microorganisms in binary or tertiary mixtures with clove oil. The one-component emulsions (EOEO and EBEO) do not show antimicrobial effects on the investigated strains, while the ECEO emulsion has an inhibitory capacity comparable to that of the positive control for most of the investigated strains. The antimicrobial potential and the synergistic effects of the active principles of essential oils (eugenol, limonene and α-pinene) are highlighted in binary E(CEO/OEO), E(CEO/BEO) or tertiary natural preparations E(CEO/OEO/BEO). Binary emulsions E(OEO/CEO) and E(BEO/CEO) are distinguished by an ability to inhibit micellar development higher to the effect generated by the positive control (gentamicin and nystatin) which recommends their use. as complementary therapies in the treatment of various infectious diseases.

## Figures and Tables

**Table 1:** The composition of natural preparation.

Emulsions	Composition				
	CEO (mL)	BEO (mL)	OEO (mL)	Lecithin (mg)	Water (mL)
Emulsion EOEO	-	-	1	6	19
Emulsion ECEO	1	-	-	6	19
Emulsion EBEO	-	1	-	6	19
Emulsion E(BEO/CEO)	1	1	-	6	18
Emulsion E(OEO/CEO)	1	-	1	6	18
Emulsion E (BEO/OEO)	-	1	1	6	18
Emulsion E (OEO/CEO/BEO)	1	1	1	6	17

**Table 2:** Mean particle size (nm), polydispersity index (PDI), Zeta-potential (ζ-Potential) (mV) of natural preparations.

Natural Preparation	Size and Particle Homogenity			Zeta-Potential (ζ-Potential) (mV)
	Mean Particle Size (nm)	Proportion of Each Population (%)	Polydispersity Index (PDI)	
EBEO	277.3	100	0.3	−21.03
ECEO	180.6 619.1	32 68	0.6	−19.72
EOEO	320.2	100	0.4	−24.16
E(BEO/CEO)	209.4 607.8	18 82	0.5	−22.20
E(BEO/OEO)	315.5	100	0.4	−24.31
E(CEO/OEO)	327.1 624.3	5 95	0.6	−20.09
E(OEO/BEO/CEO)	292.9 611.2	37 63	0.8	−22.47

**Table 3:** The MIC (µL/100 mL) for chemical compounds (eugenol, limonene and alfa-pinene), EOs: clove (CEO), orange (OEO), bergamot (BEO) and natural preparations.

				MIC (µL/100 mL)					
Strains Component	*Streptococcus pyogenes* (ATCC 19615)	*Staphylococcus aureus* (ATCC 25923)	*Shigella flexneri* (ATCC 120022)	*Pseudomonas aeruginosa* (ATCC 27853)	*Escherichia coli* (ATCC 25922)	*Salmonella typhimurium* (ATCC 140028)	*Haemophillus influenzae* type B ATCC 100211	*Candida parapsilopsis* (ATCC 220019)	*Candida albicans* (ATCC 100231)
EOEO	140	140	140	140	140	140	140	140	140
ECEO	140	140	140	140	140	140	40	140	140
EBEO	140	140	140	140	140	40	140	140	140
E(OEO/BEO)	140	140	140	140	140	140	40	140	140
E(OEO/CEO)	40	40	40	40	40	40	40	40	40
E(BEO/CEO)	40	40	40	40	140	40	40	40	40
E(OEO/BEO/CEO)	140	40	140	40	140	40	40	40	40
OEO	2	2	2	7	2	2	2	2	2
CEO	2	2	7	2	7	2	2	2	2
BEO	7	2	2	2	2	2	2	2	2
Pinene	2	2	2	2	2	2	2	2	2
Limonene	7	2	2	2	2	2	2	2	2
Eugenol	2	2	2	2	2	2	2	2	2

The samples that had no inhibition effect, causing a mass growth of the strain are marked with the dark grey color. The light gray color represents the samples in which the MIC was found, but subsequent concentrations showed a potentiating effect, therefore the effect decreases with the concentration. The white color highlights the samples in which the MIC was determined and the effect is maintained with an increase in concentration.
